# Nuclear Lamina Dysfunction and DNA Damage as Drivers of Premature Senescence in a Human Müller Glial Cell Model of Spinocerebellar Ataxia Type 7

**DOI:** 10.3390/ijms27135714

**Published:** 2026-06-24

**Authors:** Vanessa Ruiz-Esparza-Palacios, Ian García-Aguirre, Guadalupe E. Jiménez-Gutiérrez, Nadia M. Murillo-Melo, Aranza Meza-Dorantes, Yessica S. Tapia-Guerrero, Oscar Pérez-Méndez, Jose M. Gonzalez-Meljem, Bulmaro Cisneros, Jonathan J. Magaña

**Affiliations:** 1 Department of Bioengineering, School of Engineering, Tecnologico de Monterrey, Campus Ciudad de Mexico, Mexico City 14380, Mexico; a01261500@tec.mx (V.R.-E.-P.); ian.garcia@tec.mx (I.G.-A.); nmurillo@inr.gob.mx (N.M.M.-M.); a01730904@tec.mx (A.M.-D.); oscar.perez.m@tec.mx (O.P.-M.); jmgonzalezmeljem@tec.mx (J.M.G.-M.); 2 Laboratory of Genomic Medicine, Department of Genetics, National Rehabilitation Institute-Luis Guillermo Ibarra Ibarra (INR-LGII), Mexico City 14389, Mexico; gejimenez@inr.gob.mx (G.E.J.-G.); yessicasarai@gmail.com (Y.S.T.-G.); 3 Department of Genetics and Molecular Biology, Center of Research and Advanced Studies (CINVESTAV-IPN), Mexico City 07360, Mexico

**Keywords:** spinocerebellar ataxia type 7, polyglutamine diseases, DNA damage, cellular senescence, Müller glial cells

## Abstract

Spinocerebellar ataxia type 7 (SCA7) is a hereditary disorder characterized by degeneration of the cerebellum and retina. SCA7 is caused by the expansion of a polyQ tract in the *ATXN7* gene, leading to protein misfolding, transcriptional dysregulation, and neuronal/glial degeneration. Recently, altered DNA damage response (DDR) was revealed in SCA7, which may contribute to disease pathogenesis. Impaired DDR causes DNA damage, which in turn triggers cellular senescence. Consistently, senescent cells were identified in the cerebellum Purkinje layer of an SCA7 mouse model. In this study a Müller glial model (MIO-M1) expressing normal (10Q) or expanded (64Q) ataxin-7 was utilized to ascertain whether mutant protein induces genomic instability and consequently the emergence of senescence. PolyQ ataxin-7 elicits nuclear lamina disorganization, γH2AX foci (DDR marker), micronuclei and telomere shortening, which indicate genomic instability. Furthermore, 64Q cells expressing polyQ ataxin-7 exhibited senescence hallmarks, including heterochromatin loss and increased senescence-associated β-galactosidase activity, but not p21 nor p53 expression. Instead of the senescence-associated enlargement of nucleoli, these cells exhibited nucleolar disaggregation. Together, these findings indicate that the expression of polyQ ataxin-7 disrupts the nuclear architecture, thereby inducing genomic instability. This, in turn, results in a senescence-like phenotype, a phenomenon that may contribute to glial pathogenesis.

## 1. Introduction

Spinocerebellar ataxia type 7 (SCA7) is an inherited neurodegenerative disease caused by an abnormal CAG repeat expansion in the coding region of the *ATXN7* gene, resulting in the synthesis of a polyglutamine (polyQ)-expanded ataxin 7 protein [1]. Clinically, SCA7 is characterized by progressive cerebellar ataxia, including gait ataxia, dysarthria, dysphagia, and dysmetria, together with macular degeneration that progressively leads to vision loss [2,3]. A distinctive feature of SCA7 is its remarkable genetic anticipation. Among the polyQ disorders, SCA7 exhibits one of the most pronounced anticipation phenomena, whereby intergenerational expansion of the CAG repeats, particularly during paternal transmission, resulting in dramatically earlier disease onset and increased disease severity in successive generations [4].

Although the molecular mechanisms underlying SCA7 are not yet fully understood, several pathogenic processes have been identified. Ataxin-7 is a key component of the STAGA (SPT3-TAF9-ADA-GCN5 acetyltransferase) complex, which regulates gene expression through chromatin remodeling and histone acetylation [5]. PolyQ expansion disrupts STAGA function, leading to transcriptional dysregulation [1,6]. In addition, mutant ataxin-7 undergoes progressive misfolding and aggregation in affected tissues of SCA7 patients and transgenic mouse models [6,7,8]. These nuclear aggregates sequester multiple cellular proteins involved in transcriptional regulation, RNA processing, protein quality control, and autophagy [1,9,10,11,12]. Consequently, aggregate formation has been associated with proteotoxic stress, activation of programmed cell death pathways, and progressive neurodegeneration of central nervous system cells [13,14].

Emerging evidence indicates that SCA7 involves additional mechanisms whose identification would contribute to a better understanding of the pathology. In this regard, genomic instability, a well-recognized hallmark of neurodegenerative disorders, has been implicated in SCA7. Recent studies have revealed an increase in DNA damage and the expression of DNA repair pathway genes in SCA7 cells [15,16]. The DNA damage response (DDR) pathway plays a critical role in preventing the propagation of damaged DNA into daughter cells. This is because the accumulation of such damage could either activate an apoptotic response or likely initiate cellular senescence [17,18]. In line with this, a recent study identified senescent cells in the kidney and the Purkinje layer in the cerebellum of a SCA7 mouse model [19]. The overall evidence suggests a mechanistic pathway from the accumulation of DNA damage due to a deficient DNA repair mechanism to the induction of senescence in SCA7. Therefore, it is timely to determine whether polyQ-expanded ataxin-7 elicits cellular senescence and whether this phenomenon contributes to the progress of the disease.

In this study, a human Müller glial cell line (MIO-M1) that expresses a polyQ ataxin-7 in an inducible manner [20,21,22] was utilized to ascertain whether the mutant protein exerts an impact on senescence-associated cellular processes that consequently enable these cells to acquire a senescent phenotype.

## 2. Results

To ascertain whether cellular senescence is an unrecognized mechanism contributing to SCA7 pathology, different hallmarks of senescence were evaluated in the MIO-M1 cell line. This cell line was previously engineered to express ataxin-7 in an inducible manner, bearing either a normal or a pathogenic polyQ expansion (10Q cells and 64Q cells, respectively) [20]. Supplementary Figure S1 confirms the expansion of the CAG repeat tract in the cellular model. Additionally, immunofluorescence analysis revealed the presence of abnormal ataxin-7 aggregates in 64Q cells, whereas no aggregate formation was observed in 10Q cells (Figure S1).

**Figure 1:**
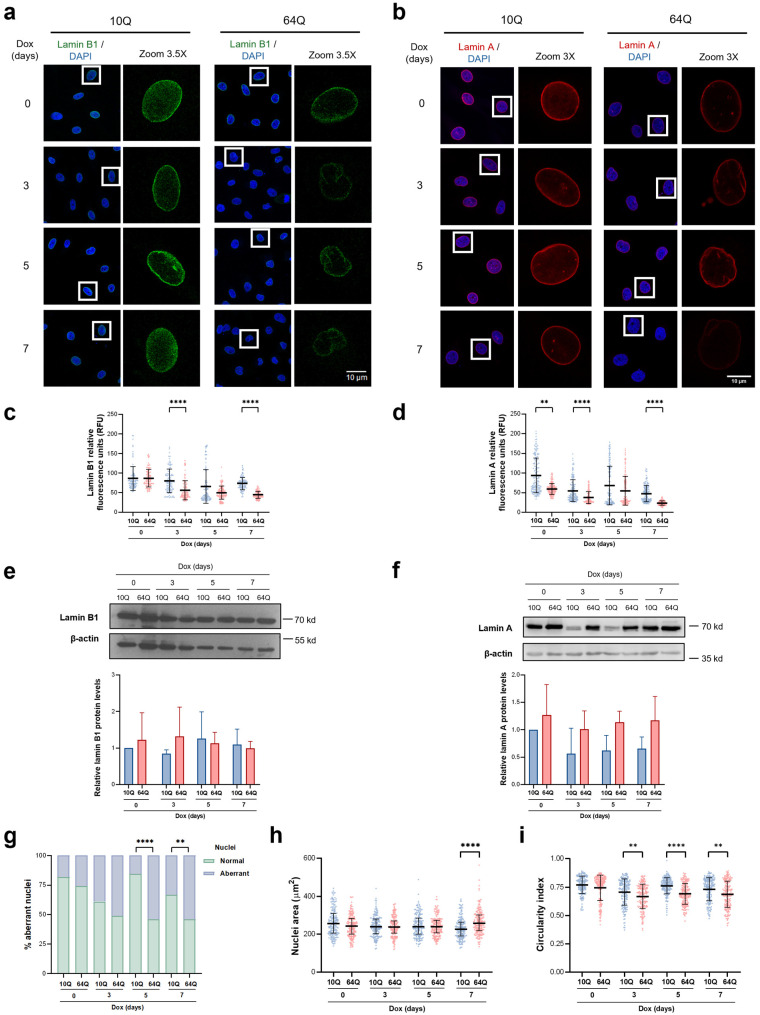
Decreased immunostaining of lamins B1 and A and altered nuclear morphology in MIO-M1 cells expressing mutant ataxin-7. 10Q and 64Q cells were induced to express ataxin-7 for the indicated time periods with 1 μg/mL dox. (**a**,**b**). The cells were then immunostained for lamin B1 (green) or lamin A (red) and counterstained with DAPI (blue) to visualize nuclei. The white square highlights the zoomed-in area. (**c**,**d**) The immunofluorescence intensity of lamin B1 (*n* = 100 nuclei per experimental condition) and lamin A (*n* = 100 nuclei per experimental condition) was determined using ImageJ. The data represents the mean ± SD from three separate experiments, and significant differences were obtained by Kruskall–Wallis test followed by Dunn’s multiple comparison test (** *p*  <  0.01; **** *p*  <  0.0001). (**e**,**f**) Cell lysates from the indicated experimental conditions were analyzed by Western blotting using primary antibodies against lamin B1, lamin A or β-actin (loading control). Relative protein levels were calculated from three independent experiments, and no significant differences were observed after Kruskal–Wallis test followed by Dunn’s multiple comparison test. (**g**) The percentage of normal and irregularly shaped nuclei were scored (*n* = 100 nuclei per experimental condition). The nuclear area (**h**) and nuclear circularity (**i**) parameters were quantified using ImageJ. The data represents the mean ± SD from three independent experiments (*n* = 180 nuclei per experimental condition), and significant differences were calculated using Kruskal–Wallis test followed by Dunn’s multiple comparison test (** *p*  <  0.01; **** *p*  <  0.0001).

The primary function of nuclear lamin is to maintain nuclear integrity, regulate gene expression, and facilitate DNA replication and repair [23]. Thus, the expression and localization of lamins B1 and A, and histone H3k9me3 (heterochromatin marker) were analyzed following the induction of 10Q or 64Q ataxin-7 protein expression for 0, 3, 5, and 7 days with 1 µg/mL of doxycycline (dox). The 10Q and 64Q cells were immunostained for lamin B1 and lamin A prior to being analyzed by immunofluorescence and confocal laser scanning microscopy (CLSM). The typical staining pattern at the nuclear periphery was observed for both lamin B1 and lamin A in untreated 10Q and 64Q cells (Figure 1a,b); however, a gradual decline in lamin B1 fluorescence intensity from day 3 to day 7 of dox treatment was evident in cells expressing 64Q ataxin-7, coinciding with the appearance of nuclear deformities (e.g., lobulations, invaginations and kidney-shaped nuclei) (Figure 1a,c). By contrast, normal nuclei morphology was observed in cells expressing 10Q ataxin-7 during the course of dox treatment. In a similar manner, the intensity of lamin A immunofluorescence decreased in a continuous fashion during the course of dox-mediated 64Q expression, coinciding with the emerging of nuclear morphology aberrations, in comparison with cells expressing 10Q ataxin-7 (Figure 1b,d). On the other hand, the protein levels of both lamin B1 and lamin A exhibited no significant changes during the induction of 10Q or 64Q ataxin-7 expression, as demonstrated by Western blotting analysis (WB; Figure 1e,f). This observed discrepancy between the results of CLSM and WB analyses indicates that the effects of mutant ataxin-7 expression were not uniform across the entire cell population, which can be attributed to the use of asynchronous cell cultures. In view of the nuclear morphometry abnormalities observed in cells expressing the mutated variant of ataxin-7, a subsequent morphometric evaluation of nuclei was conducted.

The cells that expressed 64Q ataxin-7 exhibited a significant increase in the number of aberrant nuclei on days 3, 5 and 7 of dox induction, compared to the cells expressing 10Q ataxin-7 (Figure 1g). Quantitative analysis demonstrated that the nuclear area had significantly increased in 64Q cells after seven days of induction (258.7 µm^2^) in comparison with 10Q cells (226.4 µm^2^) (Figure 1h). Furthermore, the circularity index demonstrated a progressively time-dependent deterioration of nuclear architecture in dox-treated 64Q cells, with significant differences observed at 3, 5, and 7 days (Figure 1i). This effect was particularly pronounced at day 5 (0.688 µm^2^) compared with 10Q cells (0.772 µm^2^). Collectively, these results indicate that the expression of polyQ-expanded (64Q) ataxin-7 leads to a progressive reduction in lamin B1 and lamin A immunostaining, ultimately promoting nuclear morphology alteration and probably lamina dysfunction.

**Figure 2:**
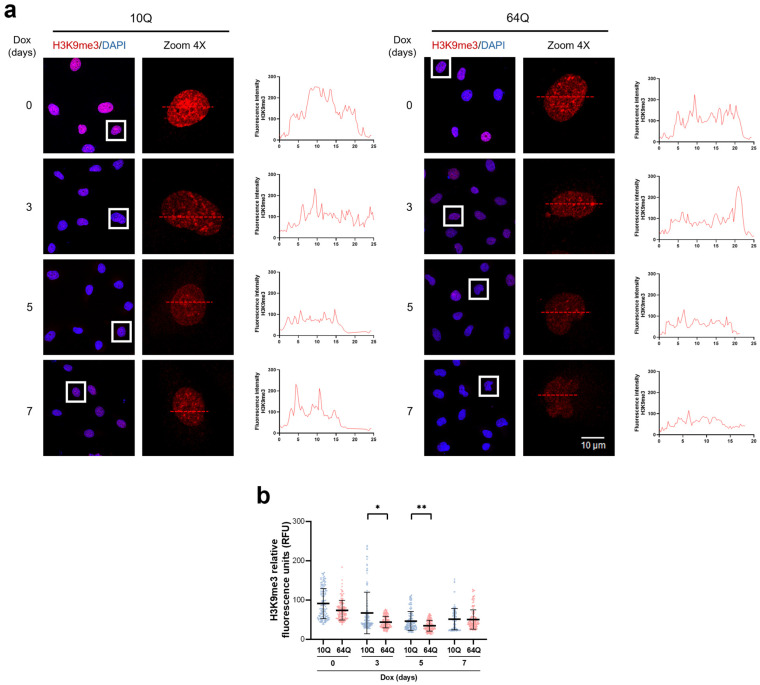
Loss of heterochromatin in MIO-M1 glial cells expressing polyQ ataxin-7. 10Q and 64Q cells were induced to express ataxin-7 for the indicated time periods. (**a**) The cells were then immunostained for H3K9me3 (red) and counterstained with DAPI (blue), to visualize the nuclei. Typical CLSM images from three separate experiments are shown with their respective line profile analysis of fluorescence (right graphs). The white square highlights the zoomed-in area. (**b**) The fluorescent intensity of H3K9me3 levels was determined (*n* = 130 nuclei per experimental condition). The data corresponds to the mean ± SD from three independent experiments and significant differences were calculated with Kruskal–Wallis test followed by Dunn’s multiple comparison test (* *p*  <  0.05; ** *p*  <  0.01).

Since proper nuclear lamina function is required for heterochromatin organization [24,25], the heterochromatin marker H3K9me3 was assessed. A decrease in H3K9me3 immunostaining was observed in both 10Q and 64Q cells from day 0 to day 5 of dox induction, with slight recovery at day 7 for both cell cultures. However, at all induction times, the fluorescence intensity of H3K9me3 was lower in 64Q cells compared to 10Q cells, as evidenced by CLSM images, line profile analysis of fluorescence, and quantification of fluorescence intensity (see Figure 2a,b). Overall, these data provide evidence that the expression of 64Q ataxin-7 is sufficient for the nuclei to lose heterochromatin organization.

### 2.1. The Expression of polyQ Ataxin-7 Evokes a DDR, the Formation of Micronuclei, γH2AX Foci and Telomere Shortening

**Figure 3:**
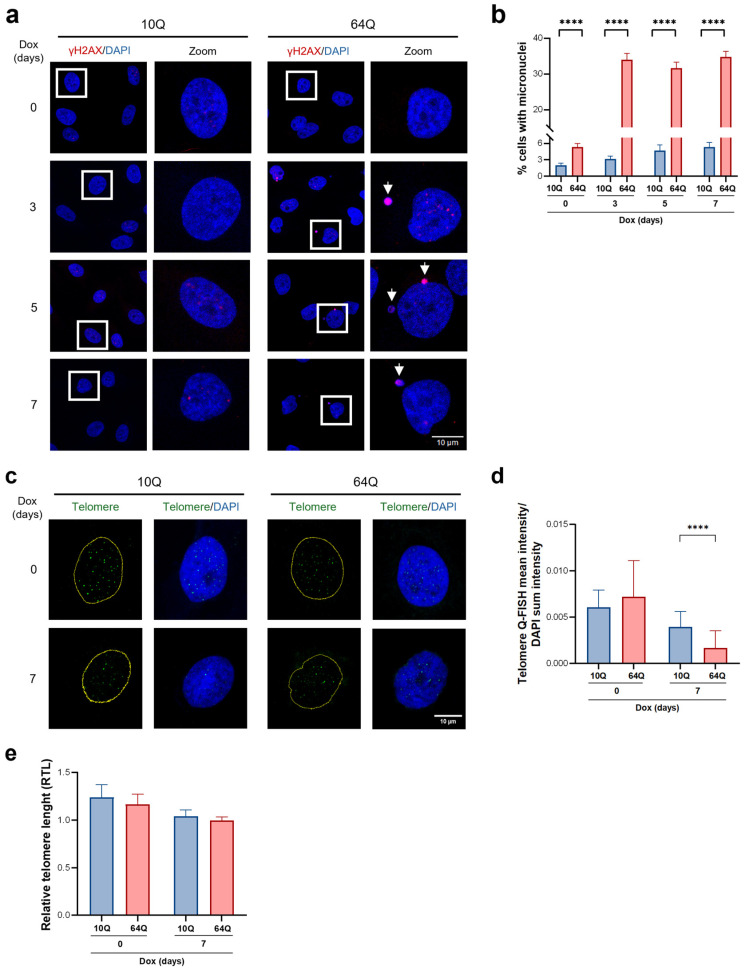
The expression of mutant ataxin-7 induces genomic instability in MIO-M1 cells. (**a**) 64Q and 10Q cells grown on coverslips were induced to express ataxin-7 proteins with 1 μg/mL dox for 0, 3, 5, and 7 days. Then, the cells were immunostained with γH2AX (red) antibodies and counterstained with DAPI (blue) to visualize the nuclei. Typical confocal images from three separate experiments are shown. The presence of micronuclei is indicated by arrows. The white square highlights the zoomed-in area. (**b**) The percentage (%) of cells with micronuclei was quantified for each experimental condition. The data corresponds to the mean ± SD (*n* = 100 cells), and statistically significant differences were calculated using Kruskal–Wallis test followed by Dunn’s multiple comparison test (**** *p*  <  0.0001). (**c**) The cell preparations were hybridized with a specific telomere probe (FISH) and stained with DAPI to visualize the nuclei. (**d**) The average of telomere fluorescent intensity was divided by the average DAPI intensity as described previously [29]. The data corresponds to the mean ± SD (*n* = 64 cells), and statistically significant differences were calculated using Kruskal–Wallis test followed by Dunn’s multiple comparison test (**** *p*  <  0.0001). (**e**) A quantitative analysis of RTL was performed by qPCR. The data corresponds to the mean ± SD from analyses of 5 independent experiments, and no significant differences were observed after Kruskal–Wallis test followed by Dunn’s multiple comparison test.

The loss of nuclear envelope structure and integrity due to lamin deficiency has been shown to result in subsequent DNA damage [24,25,26]. In line with this, it was found that 64Q cells contained immunofluorescent foci of γH2AX after three days of dox-mediated ataxin-7 expression, while 10Q cells showed some foci of γH2AX up to five and seven days of dox exposure (Figure 3a). Histone H2AX is rapidly phosphorylated at serine 139 in response to DNA double-strand breaks (DSBs) to form γH2AX [27]. On the other hand, 64Q cells exhibited a higher number of micronuclei in comparison to 10Q cells under basal conditions (Figure 3a,b). This feature of genomic instability increased significantly upon induction of polyQ ataxin-7, with a peak number of micronuclei observed at seven days of dox treatment compared with that shown by 10Q ataxin-7 expressing cells (Figure 3a,b). It is worth noting that most 64Q cells contained a single micronucleus, while less than 1% of 10Q cells exhibited micronuclei. The DDR mechanism is also triggered by the dysfunction of telomeres due to shortening or loss of capping proteins [28]. Therefore, we presumed that telomere homeostasis may be altered by polyQ ataxin-7 expression. The telomere length of 64Q and 10Q cells was assessed by quantitative fluorescence in situ hybridization (Q-FISH) at seven days of dox-mediated 64Q or 10Q ataxin-7 expression, because the former cells exhibited numerous polyQ ataxin-7 aggregates at this time point. This technique uses fluorescently labeled PNA (peptide nucleic acid) probes that bind to telomeric DNA repeats, with signal intensity corresponding directly to telomere length [29]. A subtle yet significant decrease in telomeric signal intensity was observed in 64Q cells following seven days of dox-mediated induction of polyQ ataxin-7, in comparison to 10Q cells (Figure 3d). To corroborate these findings, relative telomere length (RTL) was measured by quantitative PCR (qPCR) as previously described by Cawthon [30]. A decreasing trend in RTL that was not statistically significant was determined in 64Q cells at seven days of dox-mediated induction of polyQ ataxin-7 (Figure 3e). Collectively, these data imply that polyQ ataxin-7 expression resulted in some genomic instability features in MIO-M1 cells, including DDR evidenced by the presence of γH2AX foci, micronuclei formation, and telomere shortening.

### 2.2. 64Q Cells Exhibit Hallmarks of Senescence and Nucleolar Disaggregation

**Figure 4:**
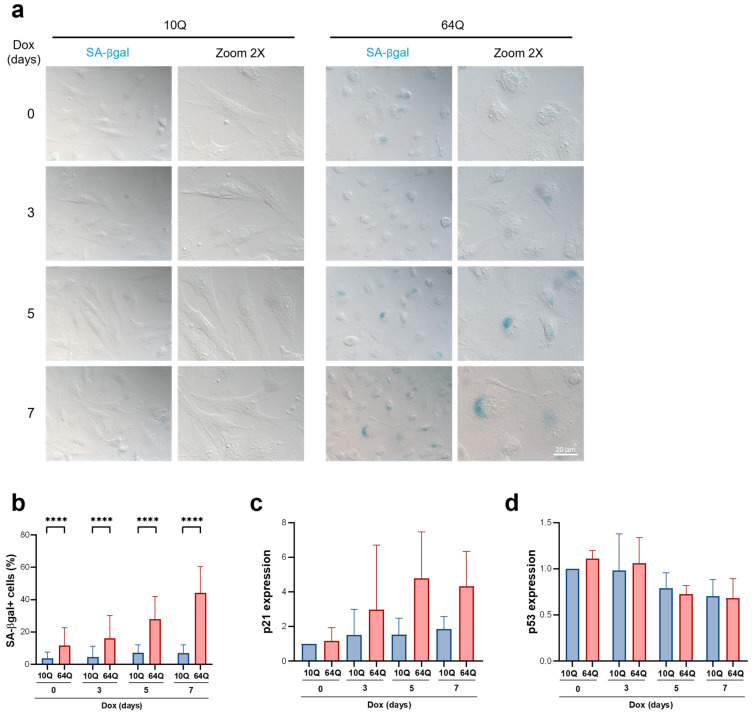
Mutant ataxin-7 expressing cells exhibited senescence features. MIO-M1 cells were induced for 0, 3, 5, and 7 days with dox. (**a**) The activity of β-galactosidase was analyzed, and representative bright field microscope images are shown. (**b**) The percentage of SA-βgal-positive cells was determined by counting the number of intensely blue-colored cells and dividing by the total number of cells. Each bar represents the mean percentage ± SD from 3 independent experiments of *n* = 500 cells, and the statistically significant differences were obtained with a Games–Howell’s test (**** *p*  <  0.0001). (**c**,**d**) Relative mRNA levels of p21 and p53 measured by real-time PCR. The data corresponds to the mean ± SD, and no statistically significant differences were observed after Kruskal–Wallis test followed by Dunn’s multiple comparison test.

To determine whether the nuclear alterations exhibited by 64Q cells, including lamina dysfunction, altered chromatin organization and genomic instability, enable them to acquire a senescent phenotype, a variety of senescent hallmarks were evaluated in these cells. Firstly, the activity of the senescence-associated β-galactosidase (SA-β-gal), a well-recognized biomarker of senescent cells, was assessed. An increase in the number of SA-β-gal-positive cells (59.5%) was observed in 64Q cells after seven days of dox-mediated polyQ ataxin-7 expression (Figure 4a), as compared to 10Q cells. Furthermore, the mRNA expression levels of the senescence-associated genes p21 and p53 were evaluated. An increasing trend in p21 mRNA expression levels, which lacked statistical significance, was observed in 64Q cells (Figure 4b) in comparison to 10Q cells. Similarly, comparable mRNA expression levels of p53 were observed between 10Q and 64Q cells during the course of dox treatment (Figure 4c).

**Figure 5:**
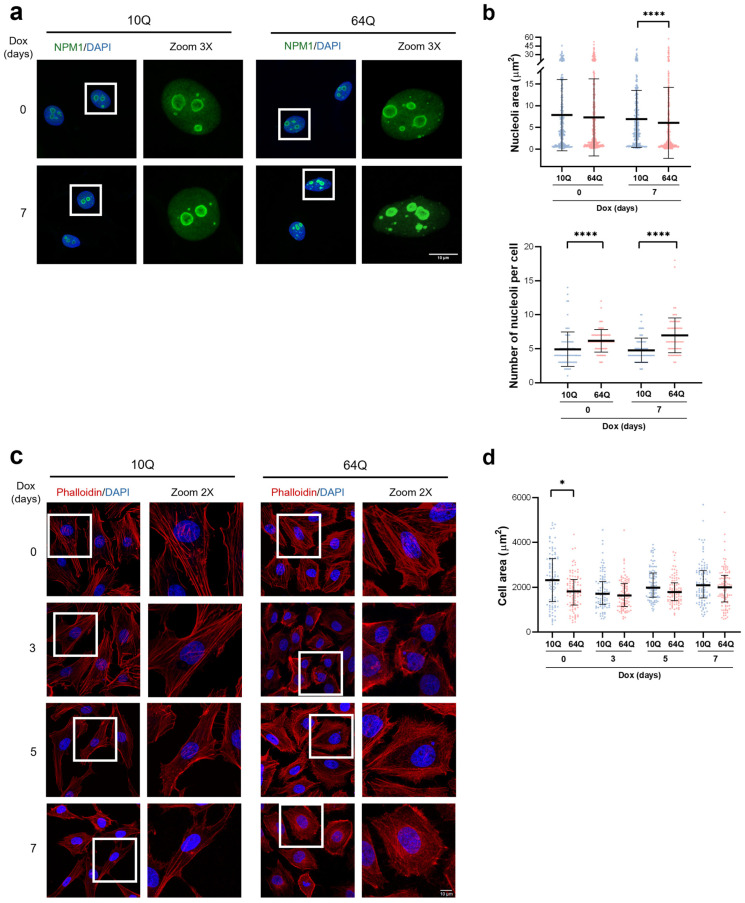
Nucleolar disaggregation is a characteristic of 64Q+ cells. MIO-M1 cells were induced for 0, 3, 5, and 7 days. (**a**) Cells were stained with NPM1 (green) and DAPI (blue). The white square highlights the zoomed-in area. (**b**) Nucleolar area was determined by outlining the area using ImageJ and then quantified. The number of nucleoli per cell was also determined. Each dot represents an individual nucleolus, and the black line represents the media ± SD from analyses of 3 independent experiments; significant differences were obtained with a Kruskal–Wallis test (**** *p*  <  0.0001). (**c**) Immunostaining with phalloidin (red) and DAPI (blue). The white square highlights the zoomed-in area. (**d**) Cells were outlined and their total cell area were quantified. Each dot represents an individual cell, and the black lines represent the mean ± SD from analyses of *n* = 95 cells; significant differences were calculated with a Kruskal–Wallis test (* *p*  <  0.05).

Given that enlarged nucleolar morphology is a hallmark of senescent cells from Hutchinson–Gilford progeria syndrome patients and healthy aged subjects [31], the size of the nucleolus in 64Q and 10Q cells was analyzed. A significant decrease in nucleolar area was found in 64Q cells at seven days of dox-mediated induction of polyQ ataxin-7, compared to 10Q cells (Figure 5a,b). Furthermore, an increase in the number of nucleoli per cell was found in 64Q cells, both before and after dox treatment, in comparison to 10Q cells (Figure 5b, bottom panel). Collectively, these data suggest that mutant ataxin-7 expression resulted in altered nucleolar area, a change that could be compatible with nucleolar disaggregation. Typically, the cellular morphology of senescent cells is flattened and enlarged [32]. Thus, the assessment of this characteristic was carried out to provide an additional parameter to determine whether 64Q cells are prone to be senescent. Although 64Q cells did not show overall differences in total cell area, they did exhibit clear morphological changes towards broader and flatter shapes, particularly at days 5 and 7 post-induction with dox, compared to 10Q cells (Figure 5c,d).

## 3. Discussion

Preliminary evidence revealed that SCA7 cells accumulate genomic damage due to deficient DNA repair response mechanisms [15,16], and an alternative study demonstrated the accumulation of senescent cells in tissues from a SCA7 mouse model [19]. In this context, the present study used an inducible MIO-M1 cell line (Tet-On system) that expresses mutant (64Q) or normal (10Q) ataxin-7 [20,21], with the aim of determining whether the expression of poly-Q ataxin-7 elicits genomic instability and consequently enables these glial cells to acquire cellular features compatible with a senescent phenotype. Interestingly, the induction of polyQ ataxin-7 expression resulted in altered organization of nuclear lamins (A and B1), impaired nuclear and nucleolar morphology, the formation of micronuclei, loss of heterochromatin, telomere alterations and the emergence of senescent cells identified by SA-β-gal staining. These data delineate a mechanistic connection from the expression of polyQ ataxin-7 that results in genomic instability and nuclear architecture alterations, to the induction of cellular senescence.

The pathophysiology of SCA7 has been predominantly conceived within the paradigm of apoptosis [13,14], in which the expansion of polyQ in ataxin-7 leads to transcriptional dysregulation, proteotoxic stress, and activation of intrinsic apoptotic pathways, resulting in progressive neuronal loss [1,33,34,35]. While this framework has been pivotal in comprehending disease progression, it does not fully reflect the molecular complexity underlying SCA7. A growing body of evidence points to the existence of additional cellular processes involved in SCA7 pathology, including epigenetic dysregulation [16,36], mitochondrial dysfunction and oxidative stress [14,37,38], and more recently, DNA repair response pathway [15,16]. We hypothesized that a mechanistic connection could exist between the altered DNA repair pathway and the presence of senescent cells in SCA7, which was timely approached in this study.

As the nuclear envelope structure dictates the functioning of a variety of processes related to senescence, including nuclear morphology, chromatin organization and genome stability, the distribution and protein levels of lamina A and B1 were analyzed upon induction of polyQ ataxin-7 expression for seven days. A significant disruption in nuclear lamina organization was identified during dox exposure, as evidenced by a reduction in lamin A and B1 immunostaining, and the presence of nuclear morphology aberrations, in cells expressing polyQ ataxin-7. These nuclear alterations were found to be associated with a loss of heterochromatin, as shown by a decrease in immunostaining of H3k9me3. The loss of heterochromatin has been shown to be a contributing factor to increased mutation rates, mitotic errors, and telomere dysfunction [39]. In line with this, expression of polyQ ataxin-7 induced micronuclei formation, which increased in frequency with longer induction times. Furthermore, γH2AX foci, which are a marker of DNA damage and telomere shortening, were evident after three and seven days of dox-mediated polyQ ataxin-7 expression. These findings are consistent with previous studies reporting increased γH2AX foci formation in both SCA7 cellular models and patient-derived fibroblasts [15,16]. We presumed that the persistence of these genomic instability markers may lead to 64Q cells to acquire a senescent state. Therefore, the assessment of senescence hallmarks in polyQ ataxin-7-expressing cells was conducted, including SA-β-Gal activity, the expression of cell cycle/senescence regulation proteins p53 and p21, and cellular and nucleolar morphologies. The nucleolus is widely recognized as a dynamic stress-sensing nuclear compartment that undergoes structural and functional remodeling in response to cellular damage and aging-related stimuli [40,41]. In senescent cells, nucleolar enlargement has been consistently reported, often accompanied by the characteristic morphological transition toward a flattened and enlarged cellular phenotype [31]. In our SCA7 cell model, the SA-β-Gal activity was found to increase in a time-dependent manner following dox-mediated expression of polyQ ataxin-7. Interestingly, SA-β-Gal staining was reported in the kidney and in the Purkinje layer of a SCA7 mouse model [19], further confirming that senescent cells accumulate in affected tissues and correlate with disease progression. However, in an unexpected manner, the nucleolar size decreased and p53 transcript levels remained unaltered upon polyQ ataxin-7 expression. On the other hand, the cellular morphology of 64Q ataxin-7 expressing cells exhibited only a tendency towards a flattened and expanded area.

Collectively, these data suggest a connection between mutant ataxin-7 expression and genome instability, as well as the acquisition of senescent features in MIO-M1 glial cells. Mechanistically, the aggregation of mutant ataxin-7 leads to a sequence of pathological events, beginning with the formation of protein aggregates that alter nuclear architecture. This disruption appears to be associated with a decrease in DNA repair efficiency, which can lead to the accumulation of DNA damage, micronuclei formation and telomere shortening. To support this hypothesis, previous studies have established a correlation between the aggregation of mutant proteins and DNA damage [42,43,44], as well as with the formation of micronuclei through unresolved DNA breaks during mitosis [45]. Furthermore, a dysfunctional DNA repair pathway has been identified in SCA7 cells [15,16]. However, the subtle telomere shortening observed in 64Q cells may be associated with oxidative stress, a recognized driver of telomere attrition [46,47]. This premise is supported by the presence of elevated ROS levels in SCA7 cell models and patient samples [14,37]. Finally, the presence of SA-β-gal positive cells upon the expression of polyQ ataxin-7 protein is consistent with the acquisition of a senescent phenotype in 64Q cells. In agreement with this, the accumulation of senescent cells in the Purkinje cell layer of the cerebellum of a SCA7 mouse model was evidenced by SA-β-gal staining [19].

It is worth noting that the polyQ ataxin-7 expressing cells failed to present upregulated levels of the cell-cycle arrest proteins p21 and p53. Although p21 expression showed a sustained upward trend throughout the experimental period, these changes did not reach statistical significance. p21 is a critical cyclin-dependent kinase inhibitor (CDKI) that binds and inhibits cyclin-CDKI complexes, specifically CDK2 and CDK1, thereby restricting S-phase entry and promoting cell cycle arrest [48,49]. It can be argued that the conventional p53-dependent DDR is not the main contributor to cell arrest during the specific time period of mutant ataxin-7 expression (1–7 days). In addition, instead of the enlarged nucleoli that are distinctive of senescent cells, nucleolar disaggregation was observed in 64Q cells, which is consistent with nucleolar stress induced by polyQ-mediated toxicity and also with lamina B1 damage or loss. Altogether the data implies that 64Q cells are undergoing a stress-induced premature senescence and require a longer period of culturing to transition from an initial stress response to a stable, p53/p21-driven arrest.

A limitation of this study is the use of asynchronous cell cultures, which may have contributed to the heterogeneous cellular responses to mutant ataxin-7 expression and could partially explain the discrepancies observed between CLSM and WB analyses. Future studies employing synchronized cell cultures may help reduce this variability and provide a more precise characterization of the temporal dynamics associated with mutant ataxin-7 expression. Additionally, although our findings reveal a strong association between nuclear lamin alterations, genomic instability, and senescence-associated features, the causal relationships among these processes remain to be fully elucidated. Therefore, studies involving longer and continuous expression of mutant ataxin-7 will be necessary to further define the mechanistic contribution of cellular senescence to SCA7 pathogenesis.

In summary, the present study delineates a pathway from nuclear structural damage to cellular senescence—like in MIO-M1 glial cells expressing polyQ ataxin-7. Mutant ataxin-7 causes the impairment of nuclear lamina and DNA damage that is not properly repaired causing the formation of micronuclei and telomere shortening, and ultimately the acquisition of a senescent-like phenotype.

## 4. Materials and Methods

### 4.1. Cell Culturing and Treatments

The generation of stable inducible MIO-M1 cell lines has been previously described [20]. The MIO-M1 cell line was obtained from Dr. Oscar Hernández-Hernández. In brief, cells were engineered using the Tet-On 3G system, in which the expression of *ATXN7* containing either 10 or 64 CAG repeats (10Q and 64Q, respectively) is induced upon the addition of dox to the culture medium. The cells were maintained at 37 °C in a humidified atmosphere with 5% CO_2_ in Dulbecco’s Modified Eagle Medium (Cat. 31600034, Gibco, New York, NY, USA) supplemented with 10% Tet System Approved fetal bovine serum (A47364-0, Gibco, New York, NY, USA), 1% penicillin–streptomycin (P4333, Sigma-Aldrich, St. Louis, MO, USA), 0.16 μg/mL puromycin (A1138-02, Gibco, New York, NY, USA), and 250 μg/mL G418 (11811064, Gibco, New York, NY, USA). For induction experiments, cells were treated with 1 µg/mL dox (D1822, Sigma-Aldrich, St. Louis, MO, USA). During the induction procedure, the culture medium was replaced at three-day intervals.

### 4.2. Indirect Immunofluorescence and Confocal Microscopy Analyses

The induction of 10Q and 64Q cells was performed using dox for varying time periods, including 3, 5, and 7 days. Forty-eight hours prior to the end of each induction period, cells were seeded onto coverslips. Subsequently, the cells were fixed with 4% paraformaldehyde (PFA) in phosphate-buffered saline (PBS) for 15 min and permeabilized with 0.2% Triton X-100 in PBS for an additional 15 min. The cells were subsequently blocked with 0.1% bovine serum albumin (BSA) (TBST), and then were incubated overnight at 4 °C with the following primary antibodies: γH2AX (ab26350, Abcam, Cambridge, UK), lamin B1 (ab16048, Abcam, Cambridge, UK), lamin A (ab26300, Abcam, Cambridge, UK), H3K9me3 (ab8898, Abcam, Cambridge, UK), and NPM1 (FC-61991, Invitrogen, Carlsbad, CA, USA). Thereafter, the cells were incubated with secondary antibodies diluted 1:200 in PBS, including fluorescein isothiocyanate (FITC) (FI-1000-1.5, Vector Laboratories, Newark, CA, USA) and DyLight 594 (DI-2594-1.5, Vector Laboratories, CA, USA). The nuclei were counterstained using Vectashield Antifade Mounting Medium with DAPI (H-1200-10, Vector Laboratories, CA, USA). The acquisition of images was performed using CLSM (ZEISS LSM 900, Oberkochen, Germany). Morphometric analyses of the nuclear area, total cell area, fluorescence intensity, and nucleolar area were performed using ImageJ software (Wayne Rasband, National Institutes of Health, USA; version 1.54p; https://imagej.net/software/fiji/ (accessed on 28 March 2026)).

### 4.3. Western Blotting

Whole-cell lysates were separated by electrophoresis on 10% SDS–polyacrylamide gels and subsequently transferred onto nitrocellulose membranes. The membranes were blocked with 3% non-fat milk in TBST for 1 h at room temperature and then incubated overnight at 4 °C with primary antibodies against lamin B1 (ab16048, Abcam, Cambridge, UK) or lamin A (ab26300, Abcam, Cambridge, UK). After primary antibody incubation, membranes were washed with TBST and then incubated with the corresponding secondary antibodies. The protein signals were detected using the Clarity™ Western ECL Substrate (Bio-Rad Laboratories, Hercules, CA, USA) according to the manufacturer’s instructions. β-Actin was utilized as a loading control and detected by incubation with an anti-actin antibody (A3854, Sigma-Aldrich, St. Louis, MO, USA).

### 4.4. Senescence-Associated β-Galactosidase (SA-β-gal) Assay

The cells were seeded on coverslips, and senescence was detected using the Senescent Cell Histochemical Staining Kit (CS0030, Sigma-Aldrich, MA, USA), according to the manufacturer’s instructions. Coverslips were mounted with 70% glycerol, and the slides were observed under a light-field inverted microscope (Nikon, Shinagawa, Tokyo, Japan).

### 4.5. Fluorescence In Situ Hybridization (FISH)

The cells were cultured on coverslips, fixed with 4% PFA in PBS for 10 min, and permeabilized with 0.2% Triton X-100 in PBS for 12 min. The samples were then subjected to RNase treatment (100 µg/mL) for a period of 20 min at 37 °C. This was followed by a series of washes with PBS and a drying process to remove any residual liquid. Thereafter, the coverslips were incubated at 80 °C for 2 h and then transferred to a hybridization buffer (20 mM Na_2_HPO_4_ [pH 7.4], 20 mM Tris [pH 7.4], 60% formamide, 10% BSA) containing 500 nm of a Cy3-labeled telomeric probe (5′ GGGTTAGGGTTAGGGTTA 3′; F1002, PNA Bio Inc., Thousand Oaks, CA, USA). The coverslips were then incubated overnight at room temperature in the dark. The following day, coverslips were subjected to a series of washes at 60 °C as follows: two washes with 2× SSC/1% Tween-20, two washes with 1× SSC/0.1% Tween-20, and one wash with 0.5× SSC/0.1% Tween-20. All washes were performed for 10 min. The samples were then mounted using Vectashield Antifade Mounting Medium with DAPI and analyzed by CLSM. The intensity of the telomere signal was then quantified using ImageJ software. RTL was calculated as the ratio between the mean telomere fluorescence intensity and total DAPI fluorescence, as previously described [29].

### 4.6. Quantitative Real-Time PCR

Total RNA was isolated from 10Q and 64Q cells using TRIzol™ Reagent (Invitrogen, Carlsbad, CA, USA), following the manufacturer’s instructions. Complementary DNA (cDNA) was synthesized from 1 µg of total RNA using the High-Capacity cDNA Reverse Transcription Kit (Thermo Fisher Scientific, Waltham, MA, USA) according to the manufacturer’s protocol. Quantitative real-time PCR (RT–qPCR) was performed on a StepOnePlus™ Real-Time PCR System (Applied Biosystems, Foster City, CA, USA) using TaqMan™ Universal Master Mix II (Thermo Fisher Scientific, Waltham, MA, USA) under standard cycling conditions. The relative mRNA expression levels of the target genes (p21 and p53) were calculated using the 2^−ΔΔCt^ method. Gene expression values were then normalized to the endogenous control *POLR2A*.

### 4.7. Relative Telomere Length (RTL) Analysis

Genomic DNA was extracted from cell pellets using a Gentra Puregene cell kit (Qiagen, Hilden, Germany). Quantitative analysis of RTL was performed using real-time PCR, a method standardized by Cawthon [30]. This method utilizes a single copy gene (albumin) to calculate the ratio between the telomeric repeats and albumin.

### 4.8. Statistical Analysis

Statistical analyses were performed with the GraphPad Prism 8.2.1 software (San Diego, CA, USA). Following a statistical analysis to ascertain the normality of the data, which revealed non-normal distribution, a Kruskal–Wallis test was implemented to compare differences among groups. This was followed by a Dunn’s multiple comparison test with *p*-value adjustment for multiple testing. In the context of parts-of-whole analysis, the determination of significance was determined by a chi-square test. The level of significance was set at *p* < 0.05. The data were expressed as the mean ± standard deviation.

## Data Availability

The original contributions presented in this study are included in the article/Supplementary Materials. Further inquiries can be directed to the corresponding author.

## Supplementary Materials

The following supporting information can be downloaded at: https://www.mdpi.com/article/10.3390/ijms27135714/s1.
