# Supplementary material for: Nuclear Lamina Dysfunction and DNA Damage as Drivers of Premature Senescence in a Human Müller Glial Cell Model of Spinocerebellar Ataxia Type 7
Source: Int J Mol Sci. 2026 Jun 24;27(13):5714. doi: 10.3390/ijms27135714 (PMC13361445; doi:10.3390/ijms27135714)
Supplement: Supplementary file 1 [file ijms-27-05714-s001.zip › ijms-4344525-supplementary.pdf]

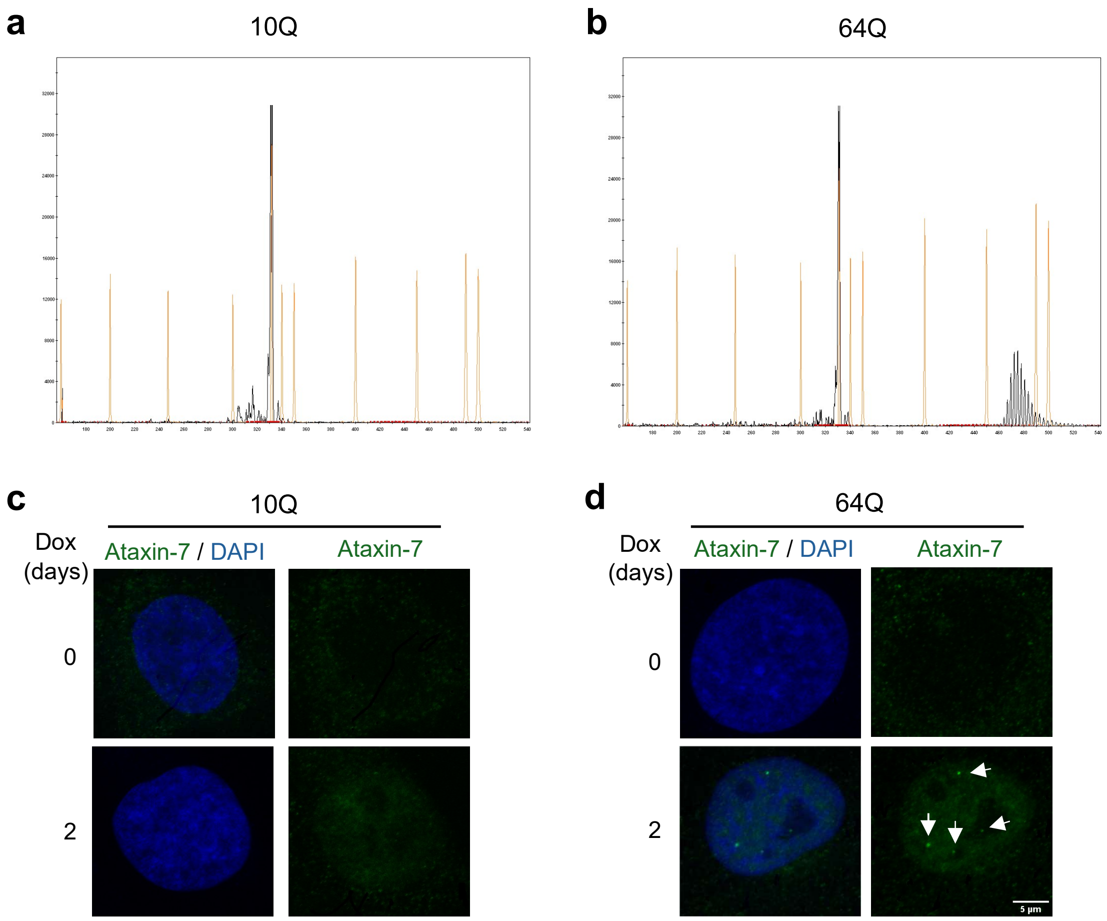

Figure S1. Validation of the inducible SCA7 cellular model. (a, b) Genotyping of ATXN7 CAG repeats by fluorescent PCR followed by capillary electrophoresis analysis. (a) Electropherogram of 10Q cells showing a homozygous genotype carrying 10 CAG repeats. (b) Electropherogram of 64Q cells carrying one normal allele with 10 CAG repeats and one expanded allele with 64 CAG repeats. (c, d) 10Q and 64Q cells were cultured for 0 or 2 days in the absence (−Dox) or presence (+Dox) of doxycycline and immunostained for ataxin-7 using an anti-ataxin-7 antibody (green), counterstained with DAPI (blue) to visualize the nuclei. (c) 10Q cells showed a diffuse ataxin-7 distribution with no evidence of nuclear aggregate formation following doxycycline treatment. (d) In contrast, doxycycline-induced 64Q cells exhibited prominent nuclear ataxin-7 aggregates (foci), confirming the expression and aggregation of mutant polyQ-expanded ataxin-7. The presence of aggregates is indicated by arrows.
